# Squamous Metaplasia in a Schwannoma: A Unique Histologic Finding

**DOI:** 10.7759/cureus.62720

**Published:** 2024-06-19

**Authors:** John Grove, Rana Naous

**Affiliations:** 1 Pathology and Laboratory Medicine, University of Pittsburgh Medical Center, Pittsburgh, USA; 2 Pathology, University of Pittsburgh Medical Center, Pittsburgh, USA

**Keywords:** nerve sheath, histologic, metaplasia, squamous, schwannoma

## Abstract

Schwannomas are benign peripheral nerve sheath tumors that originate from Schwann cells and characteristically display a biphasic appearance of compact hypercellular and myxoid hypocellular areas, named Antoni A and Antoni B areas, respectively. While most schwannomas arise sporadically, they can be associated with familial tumor syndromes such as neurofibromatosis type 2 and Carney complex. Herein, we report a case of a 61-year-old female who had a schwannoma resected from her upper extremity that later revealed a focus of squamous metaplasia associated with the schwannoma, a finding that has not yet been reported in the literature. This unique finding may aid pathologists in the future when confronted with such an atypical presentation in a schwannoma.

## Introduction

Schwannomas are benign peripheral nerve sheath tumors comprised of differentiated neoplastic Schwann cells [1]. There are multiple subtypes of schwannomas, such as ancient, cellular, plexiform, epithelioid, and microcystic/reticular schwannomas [2]. Most schwannomas (around 90%) occur sporadically and are believed to occur due to the loss of function of the tumor suppressor gene merlin [3]. Certain familial tumor syndromes, such as neurofibromatosis type 2, schwannomatosis, and Carney complex, can give rise to schwannomas in 10% of cases. Schwannomas commonly occur in adults with a peak incidence between the ages of 50 and 60 years, and the most common location is identified as the upper extremities [4]. Race or gender has not been reported as a predisposing risk factor [4]. The literature reports few cases of schwannoma with heterologous or metaplastic glandular differentiation [5]. Squamous metaplasia on the other hand has never been reported so far in schwannomas. Herein, we report the first known documented case of schwannoma with focal squamous metaplasia.

## Case presentation

**Figure 1:**
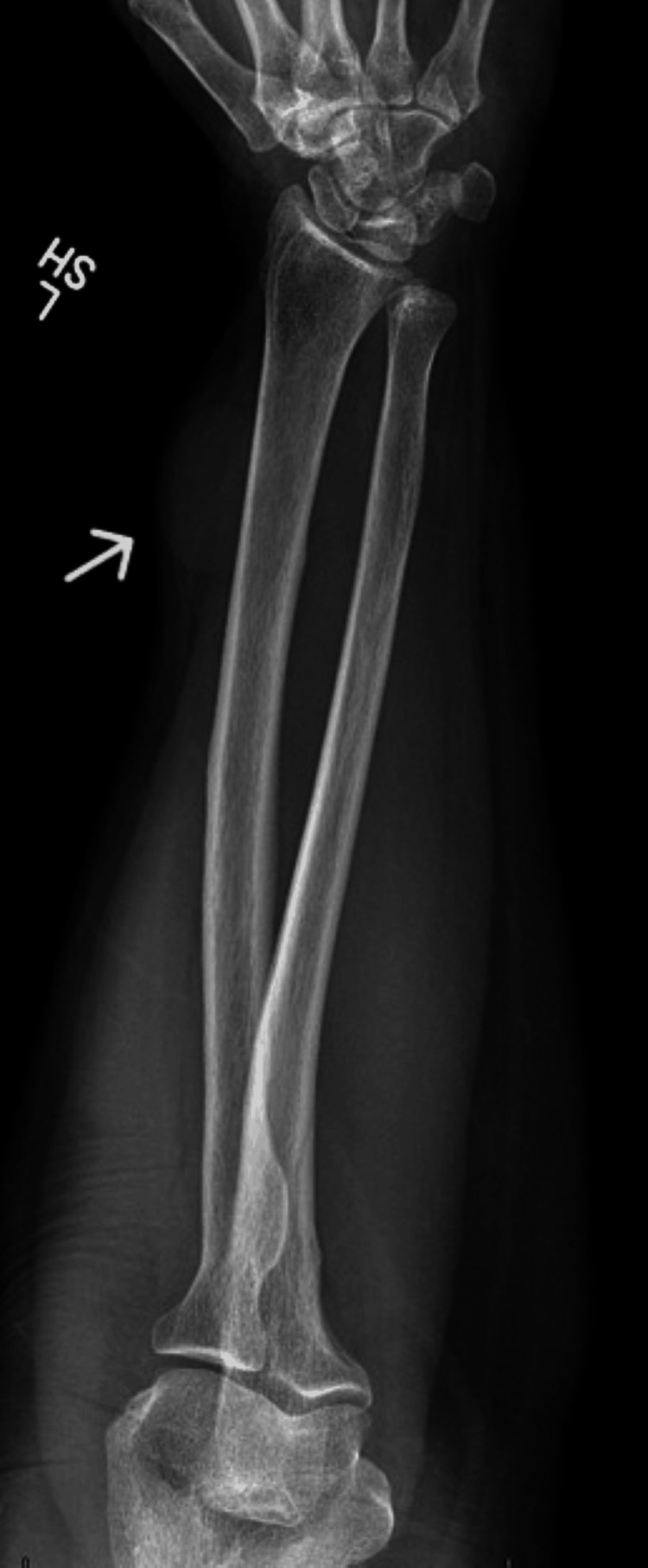
Radiologic imaging. X-ray of left forearm; arrow at mass.

A 61-year-old female presented with a left forearm mass that subsequent X-ray imaging demonstrated a left forearm mass located in the deep soft tissue without bone involvement (Figure 1). Magnetic resonance imaging (MRI) was performed at an outside institution and showed a 3.5 x 2.9 x 1.8 cm heterogeneously enhancing T2 hyperintense and T1 isointense to slightly hyperintense mass arising between the abductor pollicis longus and extensor pollicis longus muscle bellies within the distal third of the forearm and coursing along the posterior interosseous nerve. A core biopsy was performed and confirmed the mass as a schwannoma. The patient also had a history of a well-differentiated (grade IA) superficially invasive endometrioid endometrial adenocarcinoma and serous borderline tumor of the right ovary status post complete marginal excision.

**Figure 2:**
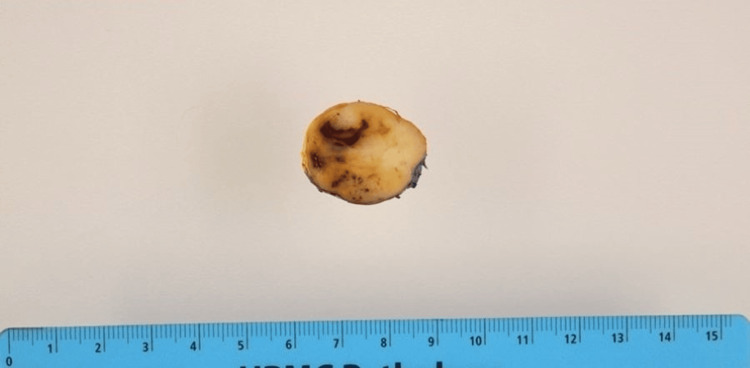
Gross image of the mass. Grossly, the tumor appears tan-white to pale-yellow with focal cystic and hemorrhagic areas.

**Figure 3:**
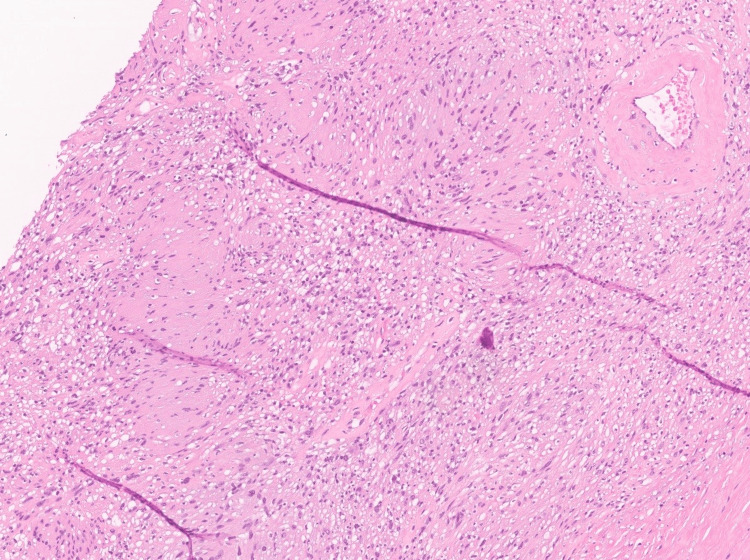
Conventional schwannoma. The tumor is thinly encapsulated (note the capsule at the right lower edge) and harbored cellular spindle cell fascicles of Antoni A with nuclear palisading (Verocay bodies) and hypocellular collagenous Antoni B areas with some vascular hyalinization and degenerative changes (hematoxylin and eosin, 10x).

**Figure 4:**
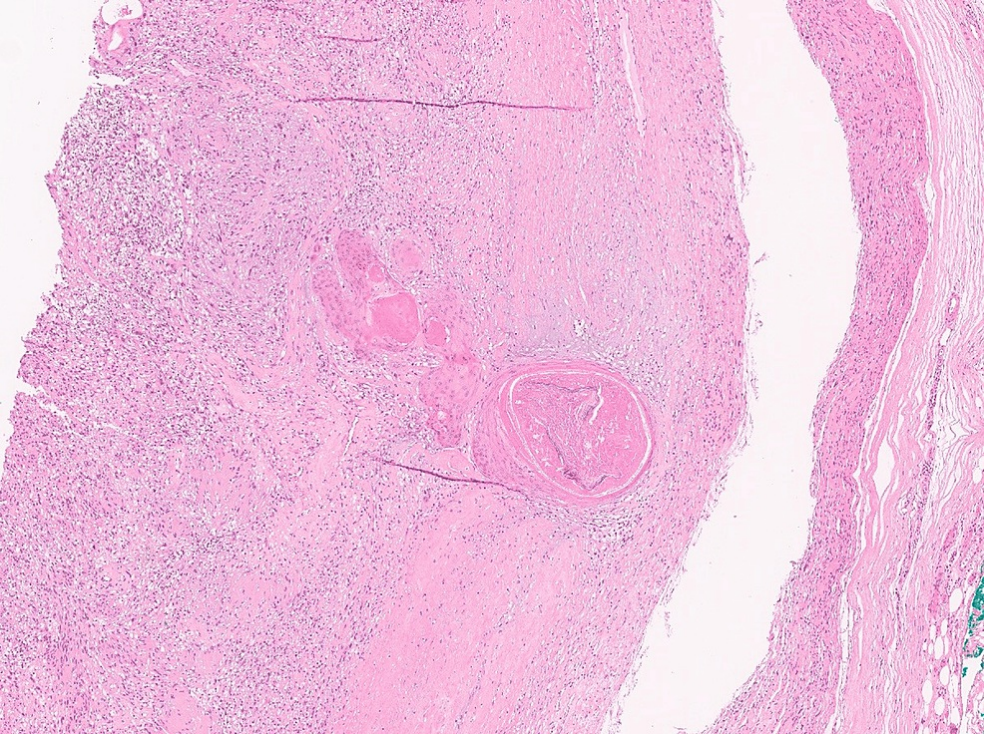
Squamous metaplasia focus within the schwannoma. Small focus on keratinizing squamous epithelium present within the background schwannoma (hematoxylin and eosin, 4x).

**Figure 5:**
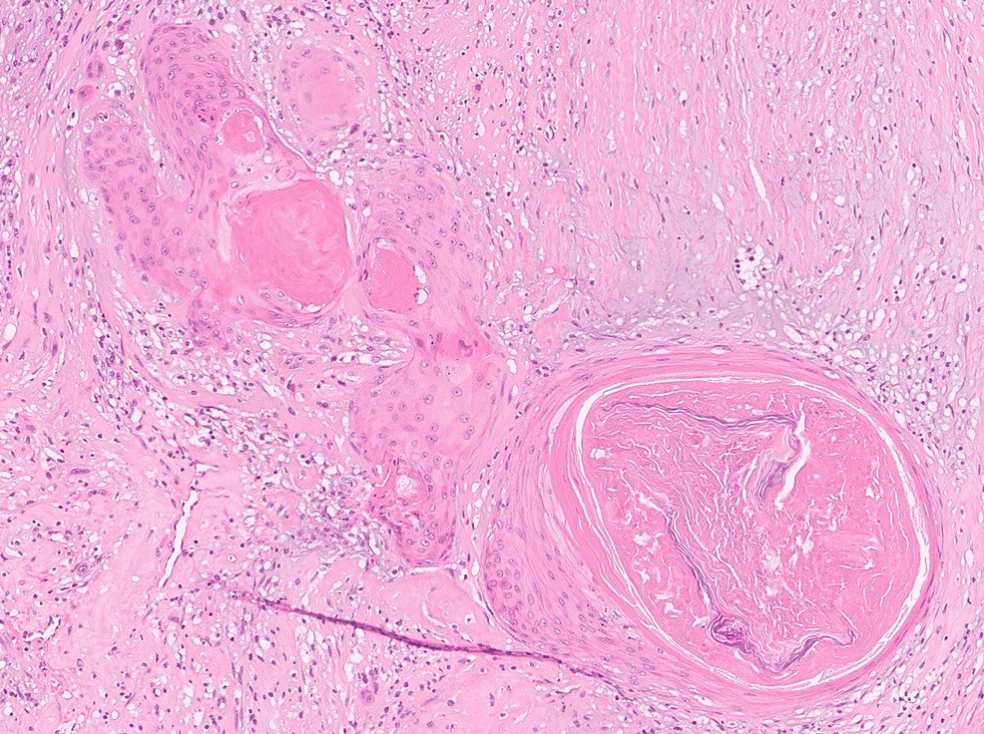
Squamous metaplasia focus within the schwannoma. The squamous epithelium appears bland with no evidence of atypia (hematoxylin and eosin, 10x).

**Figure 6:**
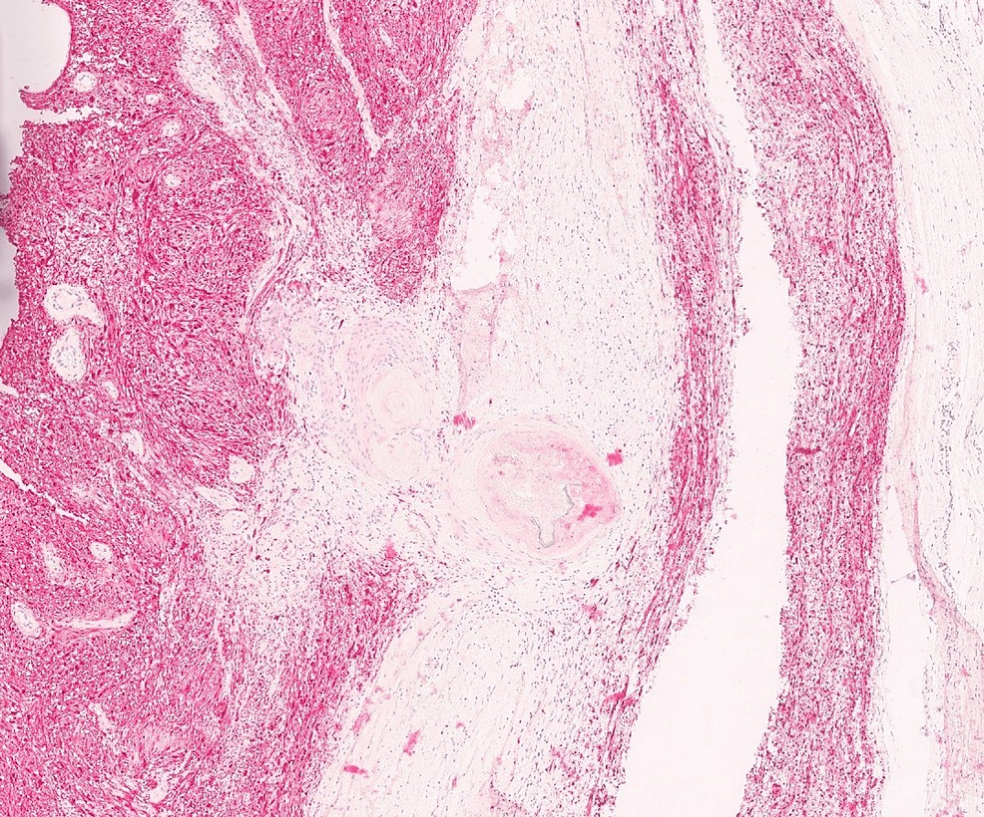
S100 immunostain. S100 immunostain is positive in the background schwannoma with sparing of the squamous epithelium focus (S100 immunostain, 4x).

**Figure 7:**
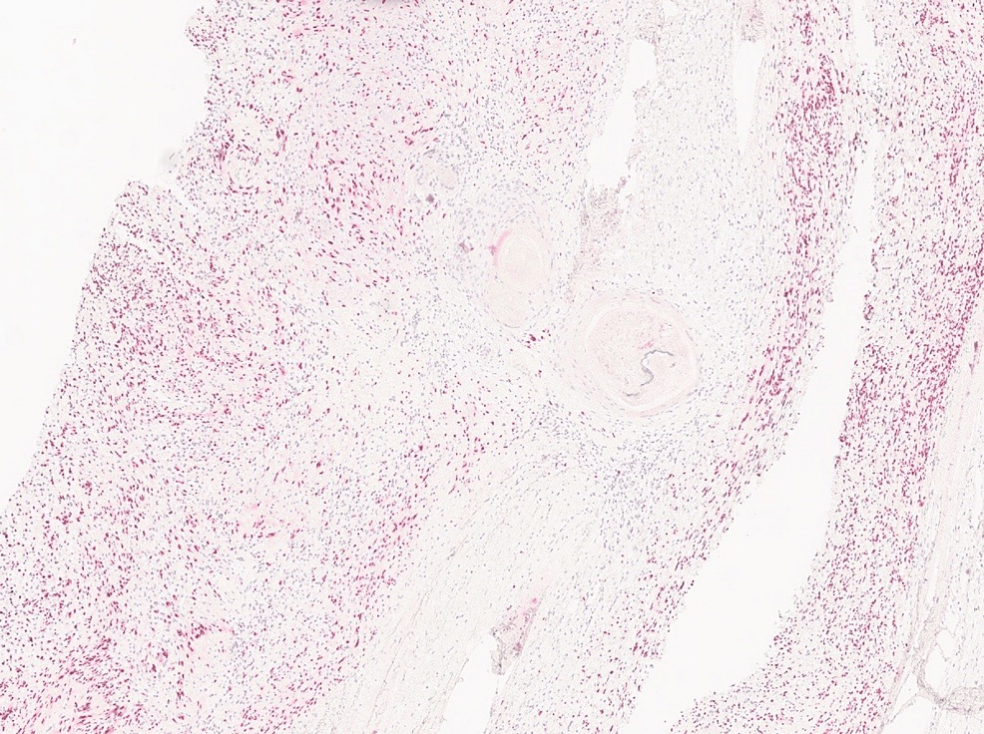
SOX10 immunostain. SOX10 immunostain is positive in the background schwannoma with sparing of the squamous epithelium focus (SOX10 immunostain, 4x).

As part of the therapeutic management, the patient underwent an uncomplicated resection of the forearm mass. Grossly, the mass measured 2.9 cm in greatest dimension and had a tan-white to pale-yellow, focally cystic to hemorrhagic appearance (Figure 2). Surgical margins were clear. Microscopically, the tumor was encapsulated, and the morphologic features were compatible with a schwannoma whereby cellular spindle cell fascicles of Antoni A with nuclear palisading and hypocellular collagenous Antoni B areas with some scattered degenerative changes were appreciated (Figure 3). Additionally, a small 2 mm focus of bland keratinizing squamous epithelium was seen within the schwannoma (Figures 4, 5). Immunohistochemical stains were positive for S100 (Figure 6) and SOX-10 (Figure 7) while HMB-45, Melan-A, and PAX-8 were negative. This unexpected epithelial focus was best categorized as focal metaplastic squamous differentiation within the schwannoma, a finding that has not been reported in the literature on schwannomas. The significance of this finding has yet to be determined.

## Discussion

Schwannomas are slow-growing benign peripheral nerve sheath tumors that typically present as asymptomatic masses or incidental findings on imaging. Medical history, such as prior radiation therapy, and the inheritance of a familial tumor syndrome, such as neurofibromatosis, are the most commonly documented medical risk factors for the development of a schwannoma. Histologically, schwannomas typically display a spindled morphology with classic hypercellular and hypocellular features, referred to as Antoni A and Antoni B areas, nuclear palisading around fibrillary process, otherwise known as Verocay bodies [6], and some degenerative changes [7].

While rare, the risk of malignant transformation in a schwannoma is estimated at <1% to 5% [8,9]. The risk of malignant transformation is approximately 10 times higher in patients with prior radiation therapy [9,10]. Additionally, epithelioid schwannomas have been reported to rarely transform into epithelioid malignant peripheral nerve sheath tumors (MPNST) [11].

In this case, a focus of squamous metaplasia was identified in a schwannoma, a histologic finding that has not been reported in the literature. Given the bland morphology of the squamous epithelial focus and the absence of glandular elements associated with it, along with the very low likelihood of a well-differentiated (grade IA) superficially invasive endometrioid endometrial adenocarcinoma to metastasize, we concluded that this squamous focus was unrelated to the patient’s endometrial carcinoma. Additionally, there are no reported incidences in the literature of carcinomas metastasizing to a schwannoma. We also entertained the possibility of the squamous focus representing entrapped native tissue; however, the deep location of the mass, being intramuscular, and the absence of any biopsy-site changes in the vicinity of the squamous epithelial focus would argue against such a hypothesis. An intramuscular epidermal inclusion cyst, albeit being in the differential diagnosis, was considered unlikely, given the absence of well-defined cystic features in the squamous epithelial focus and the rarity of such entity with only four cases of intramuscular epidermal inclusion cysts reported thus far in the literature [12]. In our opinion, this incidental epithelial focus is best considered a metaplastic focus of squamous differentiation arising within a schwannoma, an observation that has not been documented before.

The exact mechanism underlying squamous metaplasia in schwannomas remains unknown. Potential causes include chronic irritation or inflammation, leading to squamous differentiation of Schwann cells, or the presence of multipotent stem cells within the tumor capable of differentiating into squamous epithelial cells under the presence of certain stimuli. Molecular and genetic studies would allow investigators to elucidate the pathways involved in this rare phenomenon.

As the clinical significance of squamous metaplasia within schwannomas remains unknown due to limited available data, how it affects the diagnosis, prognosis, or treatment cannot be decided. While most schwannomas are asymptomatic and benign, the presence of squamous metaplasia could potentially complicate the diagnosis and treatment. In the presence of such focus, the differential diagnosis may include MPNST with heterologous squamous differentiation, melanoma with heterologous squamous differentiation, myoepithelial tumor with focal squamous differentiation, and squamous cell carcinoma metastasizing to a schwannoma. The characteristic tumor morphology with Antoni A and Antoni B features, the tumor immunophenotype with strong positivity for S100 and SOX10 [13], and absent melanoma-specific markers (HMB45 and Melan A) along with the bland tumor morphology and absence of cytologic atypia within the squamous focus aided in excluding all the aforementioned entities and supported the diagnosis of schwannoma with focal squamous metaplasia. Practicing pathologists could utilize these findings should they encounter such a unique entity.

## Conclusions

Schwannomas with unique histologic findings are rarely reported in the literature. Here, we present a unique case of schwannoma with focal squamous metaplasia, a finding not yet reported in the literature. Squamous metaplasia in schwannoma is a rare phenomenon, and albeit being of uncertain significance can present as a potential diagnostic pitfall. Careful morphologic examination, a complete immunohistochemical workup, and clinical/radiologic correlation are important diagnostic tools in reaching the right diagnosis.
